# Hypomethylation and Genetic Instability in Monosomy Blastocysts May Contribute to Decreased Implantation Potential

**DOI:** 10.1371/journal.pone.0159507

**Published:** 2016-07-19

**Authors:** Blair R. McCallie, Jason C. Parks, Alyssa L. Patton, Darren K. Griffin, William B. Schoolcraft, Mandy G. Katz-Jaffe

**Affiliations:** 1 National Foundation for Fertility Research, Lone Tree, Colorado, 80124, United States of America; 2 Colorado Center for Reproductive Medicine, Lone Tree, Colorado, 80124, United States of America; 3 School of Biosciences, University of Kent, Canterbury, CT2 7NJ, United Kingdom; Justus-Liebig-Universität, GERMANY

## Abstract

DNA methylation is a key epigenetic mechanism responsible for gene regulation, chromatin remodeling, and genome stability, playing a fundamental role during embryonic development. The aim of this study was to determine if these epigenetic marks are associated with chromosomal aneuploidy in human blastocysts. Surplus, cryopreserved blastocysts that were donated to research with IRB consent were chosen with varying chromosomal aneuploidies and respective implantation potential: monosomies and trisomies 7, 11, 15, 21, and 22. DNA methylation analysis was performed using the Illumina Infinium HumanMethylation450 BeadChip (~485,000 CpG sites). The methylation profiles of these human blastocysts were found to be similar across all samples, independent of chromosome constitution; however, more detailed examination identified significant hypomethylation in the chromosome involved in the monosomy. Real-time PCR was also performed to determine if downstream messenger RNA (mRNA) was affected for genes on the monosomy chromosome. Gene dysregulation was observed for monosomy blastocysts within significant regions of hypo-methylation (*AVEN*, *CYFIP1*, *FAM189A1*, *MYO9A*, *ADM2*, *PACSIN2*, *PARVB*, and PIWIL3) (P < 0.05). Additional analysis was performed to examine the gene expression profiles of associated methylation regulators including: DNA methyltransferases (*DNMT1*, *DNMT3A*, *DNMT3B*, *DNMT3L*), chromatin modifying regulators (*CSNK1E*, *KDM1*, *PRKCA*), and a post-translational modifier (*PRMT5*). Decreased RNA transcription was confirmed for each *DNMT*, and the regulators that impact *DNMT* activity, for only monosomy blastocysts (P < 0.05). In summary, monosomy blastocysts displayed hypomethylation for the chromosome involved in the error, as well as transcription alterations of associated developmental genes. Together, these modifications may be contributing to genetic instability and therefore be responsible for the limited implantation potential observed for full monosomy blastocysts.

## Introduction

During reproduction, an embryo receives one set of chromosomes from the sperm, and one set from the oocyte, resulting in a complete set of 23 chromosome pairs. Errors during meiotic or mitotic cell division can lead to extra or missing chromosomes, termed aneuploidy, which is the leading cause of miscarriage, stillbirth, and congenital birth defects [1]. The most significant risk factor for an aneuploid conception is advanced maternal age. In fact, 35–50% of oocytes from women aged 35–39 will have chromosomal aneuploidies and this will climb to over 80% once a woman reaches 45 years of age [2]. Aneuploidy can occur for any chromosome with the highest proportion observed in conception belonging to the smaller sized chromosomes (15–22) [3]. Only a fraction of full aneuploidies, specifically trisomies 13, 18, 21, XXY, and XYY, will develop past the first trimester and may even result in a live birth [4]. Nevertheless, even the vast majority (>95%) of these trisomies will perish in utero. This is in contrast to full monosomies which almost never implant or result in an ongoing pregnancy. Turner Syndrome (XO) is the only exception and the only full monosomy known to reach term. Partial fetal autosomal monosomies are observed during clinical pregnancy, where only a portion of a chromosome is missing, and these imbalances can lead to various phenotypes depending on the chromosome involved, the size of the absent chromosome, and which genes are impacted [5].

DNA methylation is a biochemical process that plays an important role in regulating gene expression without altering the underlying DNA sequence and involves the addition of a methyl group to a cytosine in a CpG dinucleotide. This is established either *de novo*, by DNA methyltransferases *DNMT3A*, *DNMT3B*, *and DNMT3L*, or during replication by the maintenance methyltransferase *DNMT1* [6]. Appropriate methylation is essential for both normal cell differentiation and development [7, 8]. Methylation is involved in chromatin structure which is responsible for proper chromosome segregation during cell division as well as regulating gene expression [9]. Global epigenetic reprogramming begins in the early embryo with DNA demethylation occurring post fertilization through to the blastocyst stage. This process is essential for the establishment of embryonic gene expression patterns during re-methylation which is required for implantation and ongoing fetal development [8]. Only imprinted genes escape demethylation to preserve their exclusive parent-of-origin-specific gene expression profiles [10]. DNA methylation is also shown to be vital in the maintenance of X chromosome inactivation which is crucial for female embryos due to the presence of two X chromosomes [11]. It is well known that disturbances during these methylation processes can result in developmental delays and/or embryo death. Loss of *Dnmt1* activity results in significantly lower DNA methylation levels, as well as impaired implantation and embryo development in mice [12]. *Dnmt3a* mutant mice develop to term but are runted and die at around 4 weeks of age while *Dnmt3b* mutant mice have no viable births as their embryos are found to have multiple developmental defects [13]. *Dnmt3l* interacts with Dnmt3a and 3b and has been shown in mice to play an important role in the regulation of genomic imprinting and embryonic development [14].

Given the importance of DNA methylation and chromosome constitution to healthy fetal/embryonic development, the aim of this study was to investigate the association between methylation, the molecular processes involved in establishing methylation, and chromosomal aneuploidies. Results revealed that trisomy blastocysts had similar methylation profiles to their euploid counterparts. In contrast, monosomy blastocysts were hypomethylated for the chromosome involved in the error and displayed altered expression of developmental genes and *DNMTs*, which could be contributing to their overall compromised implantation potential.

## Methods and Materials

### Blastocysts

Surplus, cryopreserved blastocysts (n = 316) from the Colorado Center for Reproductive Medicine were donated for research with written IRB consent, including blastocysts donated from donor oocyte cycles. This study was approved by HCA-HealthONE (study #231587) and Western Institutional Review Board (study #1145350). All blastocysts were viable and morphologically similar, graded as high quality expanded blastocysts (≥ 3BB) on day 5 of embryonic development using the Gardner and Schoolcraft system [15]. Blastocysts underwent trophectoderm biopsy for comprehensive chromosome screening prior to vitrification using the cryotop method as previously described [16]. The control group consisted of euploid, day 5 blastocysts produced from donor oocyte IVF cycles with no male factor infertility. Specific aneuploidies were chosen based on their differing implantation potential and included chromosomes 7, 11, 15, 21, and 22. Trisomies 7 and 11 are most likely to result in implantation failure; trisomies 15 and 22 are able to implant however will always result in miscarriage; and trisomy 21 embryos will implant but result in either miscarriage, still birth, or live birth.

### DNA Lysis and Methylation Analysis

After warming, blastocysts (n = 230) were lysed using the EZ DNA Methylation-Direct^™^ Kit (Zymo Research, Irvine CA). Briefly, pools of 10 re-expanded blastocysts, with 2–3 biological replicates per group, were washed through a series of PBS washes before being lysed in a digestion buffer containing 20ug Proteinase K in a 20ul final volume. Samples were incubated at 50°C for 20 minutes and then stored at -80°C. All 20ul of each sample were bisulfite converted by adding 130ul of CT Conversion Reagent and incubated at 98°C for 8 minutes and 64°C for 3.5 hours. Samples were then purified on the Zymo-Spin^™^ IC Column according to manufacturer’s protocol and eluted in 10ul of M-Elution Buffer. 500ng of each sample were amplified, fragmented, and hybridized to the Infinium HumanMethylation450K BeadChip (Illumina, San Diego CA). GenomeStudio Methylation Module 1.0 software (Illumina) was used for image processing and to perform normalization and differential methylation analysis. Normalization was performed using both normalization control probes as well as background subtraction. Methylation beta values were then determined for each sample which estimate the methylation level of the CpG locus using the ratio intensities between methylated and unmethylated alleles. A value of “0” represents no methylation and a value of “1” indicates full methylation. DiffScore was calculated using the Illumina Custom Model to determine significance at P < 0.05. Variance was estimated across replicate samples.

### RNA Isolation, Reverse Transcription, and Real-Time PCR

RNA was either isolated using the PicoPure RNA Isolation Kit (Life Technologies, Grand Island, NY) or lysed and deoxyribonuclease treated using the Taqman^®^ Gene Expression Cells-to-Ct^™^ Kit (Life Technologies). For primer-based assays, warmed blastocysts (n = 50) were washed through ice-cold phosphate buffered saline (PBS) containing bovine serum albumin (BSA) prior to being transferred into 10ul of Extraction Buffer. RNA was then purified from individual blastocysts (PicoPure) according to manufacturer’s protocol with minor modifications [17]. RNA quantity and quality were assessed using the NanoDrop^®^ Spectrophotometer ND-1000 (Thermo Scientific, Wilmington DE) before being reverse transcribed using the High Capacity cDNA Archive Kit (Life Technologies) where 20ul of a master mix was combined with all 20ul of the purified RNA and incubated according to protocol.

For Taqman^®^ assays, warmed blastocysts (n = 36) were washed as previously mentioned prior to being individually transferred into 10ul of Lysis Solution containing DNase I (Cells-to-Ct^™^) and incubated at room temperature for 8 minutes. 1ul of Stop Solution was added to each sample and incubated at room temperature for 2 minutes. Samples were reverse transcribed with 30ul of master mix and 10ul of RNA lysate. cDNA was then amplified by using 37.5ul of Taqman^®^ PreAmp Master Mix containing 0.05X of each Taqman^®^ probe with 12.5ul of the cDNA under the following thermal cycling conditions: 95°C for 10 minutes and 12 cycles at 95°C for 15 seconds and 60°C for 4 minutes.

Primer-based quantitative real-time PCR (qPCR) was performed using the ABI 7300 Real-Time PCR System (Life Technologies) by combining 5ul of diluted cDNA (1:4) with 7ul water, 12.5ul SYBR Green PCR Master Mix (Life Technologies) and 0.5ul of 5uM primer pool. After a 10 minute incubation at 95°C, amplification occurred for 40 cycles at 95°C for 15 seconds and 60°C for 1 minute, followed by a dissociation stage. Standard curves were calculated for each gene by performing 10-fold serial dilutions of reference RNA (Agilent, Santa Clara CA). Expression of 8 genes of interest were analyzed in duplicates (*AVEN*, *CYFIP1*, *FAM189A1*, *MYO9A*, *ADM2*, *PACSIN2*, *PARVB*, and *PIWIL3*) relative to an internal house-keeping gene, *PPIA*, which had the most consistent expression across all samples (Table 1). Negative controls were performed for each gene and all remained unamplified with Ct values at 40.

**Table 1:** Primer information and qPCR efficiencies for chromosomes 15, 22, and housekeeping genes.

Gene	Accession #	Slope	Amplicon GC	Chromosome	Primer Sequence (3'-5')
		Intercept	Amplicon Length		
		R^2^			
AVEN	NM_020371	-3.29	46%	15	F: AAGAGCTGGAAGACTGGTTGGA
		28.37	98		R: TATGCCCACCTGCCGTTAG
		0.99			
CYFIP1	NM_014608	-3.51	51%	15	F: ACGACCACTCAGCGTACAAGAG
		25.71	78		R: TCTGCGATTCCTGGATGGA
		0.99			
FAM189A1	NM_015307	-3.07	62%	15	F: GGGACACCCAGGATGATCTG
		31.47	97		R: GGAAATGCAATCCCCAAAGAG
		0.99			
MYO9A	NM_006901	-3.26	45%	15	F: CAATACACTGGAACGCCTCATC
		27.11	92		R: ACACAATGGCCAAAGCATTAGC
		0.99			
ADM2	NM_001253845	-2.97	59%	22	F: GAGCCTAAACACCCTGAAATTGTG
		28.92	88		R: TCTCTGAAGCGCTTAGCATCTG
		0.99			
PACSIN2	NM_001184970	-3.57	54%	22	F: AAGCCCTGGGCCAAGAAG
		26.99	59		R: GCTGCATGGTGGGCTTTC
		0.99			
PARVB	NM_001003828	-3.45	62%	22	F: TCTCTGGCCATGCACTTCAG
		25.31	65		R: ACCACCACCTGCACCGTTAC
		0.99			
PIWIL3	NM_001008496	-1.51	44%	22	F: AAAGAGCGGAGAGTGGAATGG
		32.88	91		R: ACGTGGGCGTGAGTTCTTTG
		0.95			
PPIA	NM_021130	-4.81	51%	7	F: GCTTTGGGTCCAGGAATGG
		21.52	59		R: TTGTCCACAGTCAGCAATGG
		0.96			

Taqman^®^ qPCR was performed by combining 4ul of diluted pre-amplified product (1:5) with 5ul nuclease-free water, 10ul Taqman^®^ Gene Expression Master Mix, and 1ul Taqman^®^ probe. This was run on the ABI7900HT Fast Real-Time PCR System (Life Technologies) at 95°C for 10 minutes, and 40 cycles at 95°C for 15 seconds and 60°C for 1 minute. Standard curves were calculated as previously described and expression of 8 genes of interest were analyzed in duplicates (*DNMT1*, *DNMT3A*, *DNMT3B*, *DNMT3L*, *CSNK1E*, *KDM1*, *PRKCA*, *and PRMT5*) relative to an internal house-keeping gene, *RPL19*, which had the most consistent expression across all samples (Table 2). Negative controls were also performed for each Taqman^®^ assay and all were found to be unamplified.

**Table 2:** Genes involved in DNA methylation processes (including two housekeeping genes) and qPCR efficiency information (Taqman^®^ assays; Life Technologies).

Gene	Entrez ID / Catalog #	Slope	Chromosome	Function
		Intercept		
		R2		
DNMT1	1786 / Hs00154749_m1	-3.46	19	Maintanence methyltransferase
		27.92		
		0.99		
DNMT3A	1788 / Hs01027166_m1	-3.48	2	*de novo* methlytransferase
		31.02		
		0.99		
DNMT3B	1789 / Hs00171876_m1	-3.35	20	*de novo* methlytransferase
		29.97		
		0.99		
DNMT3L	29947 / Hs01081364_m1	-2.73	21	In-active methyltransferase essential for the
		34.00		function of DNMT3A and DNMT3B
		0.95		
CSNK1E	1454 / Hs00266431_m1	-3.95	22	Post-translational regulation
		28.02		
		0.99		
KDM1	23028 / Hs01002741_m1	-3.76	1	Post-translational regulation
		29.90		
		0.99		
PRKCA	5578 / Hs00925193_m1	-4.13	17	Post-translational regulation
		30.52		
		0.97		
PRMT5	10419 / Hs01047356_m1	-3.77	14	Chromatin modifying protein
		29.17		
		0.99		
PPIA	5478 / Hs04194521_s1	-3.71	7	Housekeeping
		24.43		
		0.99		
RPL19	6143 / Hs01577060_gH	-3.53	17	Housekeeping
		25.18		
		0.99		

Data normalization and analysis were performed using REST 2009 software (Qiagen, Valencia CA). REST software uses the correction for exact PCR efficiencies with mean crossing point deviations between sample and control groups to determine an expression ratio that is tested for significance by a Pair Wise Fixed Reallocation Randomization Test. Significance was defined as P < 0.05.

## Results

### Global Methylation Analysis

Analysis of the blastocyst methylome for monosomies 7, 11, 15, 21, and 22, as well as trisomies 7, 11, 15, 21, and 22, compared to control blastocysts, was performed using the Illumina Infinium HumanMethylation450K BeadChip. To avoid bias, groups were blinded and Illumina GenomeStudio Software was used for normalization, beta value calculations, and DiffScore determination. When analyzing the overall methylation profiles of any blastocyst group, no significant differences were observed regardless of chromosome constitution. The average beta value (0 = no methylation, 1 = full methylation) for each group was similar, ranging from 0.20 to 0.21, representing an overall hypomethylated state (Table 3). For comparison, a typical somatic cell has a beta value of around 0.5 [18]. Further examination of the methylome of each individual chromosome revealed all trisomy blastocysts, independent of which chromosome had a third copy (7, 11, 15, 21, or 22), were similar to the diploid state (Table 4). For example, the beta value of chromosome 11 in trisomy 11 blastocysts was 0.21 (Table 4, column D) and the beta value of chromosome 11 in control blastocysts was 0.22 (Table 4, column A). In contrast, all monsomy blastocysts showed a decreased methylated state for the specific missing chromosome in comparison to controls. In this case, monosomy 11 blastocysts displayed significant hypomethylation of chromosome 11 with a beta value of 0.17 (Table 4, columnE, P < 0.05) compared to chromosome 11 in either trisomy or control blastocysts which had beta values of 0.21 and 0.22 respectively. All other correctly-paired chromosomes from these aneuploid blastocysts displayed a methylation profile similar to control blastocysts (Table 4).

**Table 3:** Methylome profiles of pooled human blastocysts (n = 10 each pool with 2–3 replicates per group). Beta value reflects the level of global methylation with no variation observed in association with blastocyst chromosome constitution. (no statistical significance). Standard deviation (STDEV) was calculated for each group, reflecting low biological variability between replicates.

Group	Beta Value	STDEV
	(Avg)	
Diploid (Euploid)	0.21	0.01
Trisomy 7	0.21	0.01
Trisomy 11	0.20	0.01
Trisomy 15	0.20	0.01
Trisomy 21	0.20	0.01
Trisomy 22	0.20	0.01
Monosomy 7	0.20	0.01
Monosomy 11	0.20	0.06
Monosomy 15	0.20	0.00
Monosomy 21	0.20	0.01
Monosomy 22	0.21	0.00

**Table 4:** Methylation profiles of individual chromosomes for blastocysts with a specific chromosome constitution: A) Diploid Control, B) Trisomy 7, C) Monosomy 7, D) Trisomy 11, E) Monosomy 11, F) Trisomy 15, G) Monosomy 15, H) Trisomy 21, I) Monosomy 21, (J) Trisomy 22, and (K) Monosomy 22 (*P < 0.05).

	A	B	C	D	E	F	G	H	I	J	K
Chr	Control	+7	-7	+11	-11	+15	-15	+21	-21	+22	-22
1	0.19	0.19	0.19	0.18	0.18	0.18	0.18	0.18	0.18	0.18	0.19
2	0.22	0.22	0.22	0.20	0.20	0.21	0.22	0.21	0.22	0.22	0.23
3	0.22	0.22	0.22	0.21	0.21	0.20	0.21	0.21	0.21	0.21	0.22
4	0.23	0.23	0.23	0.22	0.23	0.22	0.22	0.21	0.23	0.22	0.23
5	0.21	0.21	0.21	0.20	0.21	0.20	0.21	0.20	0.21	0.20	0.21
6	0.20	0.20	0.20	0.19	0.19	0.19	0.19	0.19	0.19	0.19	0.20
7	0.25	0.26	0.23*	0.24	0.24	0.24	0.24	0.24	0.24	0.24	0.25
8	0.24	0.24	0.23	0.23	0.23	0.22	0.23	0.22	0.23	0.23	0.24
9	0.21	0.21	0.20	0.20	0.20	0.20	0.20	0.19	0.20	0.20	0.21
10	0.23	0.22	0.22	0.21	0.22	0.21	0.22	0.21	0.22	0.22	0.22
11	0.22	0.21	0.21	0.21	0.17*	0.21	0.21	0.20	0.21	0.21	0.22
12	0.22	0.21	0.21	0.20	0.21	0.20	0.21	0.20	0.21	0.21	0.21
13	0.24	0.24	0.23	0.23	0.23	0.23	0.23	0.23	0.23	0.23	0.24
14	0.21	0.20	0.20	0.20	0.20	0.19	0.20	0.19	0.20	0.20	0.21
15	0.22	0.22	0.22	0.21	0.22	0.22	0.18*	0.21	0.22	0.22	0.22
16	0.23	0.22	0.22	0.22	0.21	0.22	0.22	0.21	0.22	0.22	0.23
17	0.20	0.20	0.19	0.19	0.19	0.19	0.19	0.19	0.19	0.19	0.20
18	0.18	0.18	0.18	0.18	0.18	0.17	0.18	0.17	0.18	0.18	0.18
19	0.18	0.18	0.17	0.17	0.17	0.17	0.17	0.17	0.17	0.17	0.18
20	0.17	0.17	0.17	0.17	0.17	0.16	0.17	0.17	0.17	0.17	0.17
21	0.23	0.22	0.22	0.21	0.22	0.21	0.22	0.22	0.18*	0.22	0.23
22	0.18	0.18	0.18	0.17	0.17	0.17	0.17	0.17	0.17	0.18	0.14*
X	0.16	0.17	0.16	0.15	0.16	0.15	0.16	0.16	0.16	0.16	0.16
Y	0.16	0.17	0.18	0.18	0.16	0.15	0.14	0.16	0.17	0.16	0.15

### Blastocyst Gene Expression

**Fig 1:**
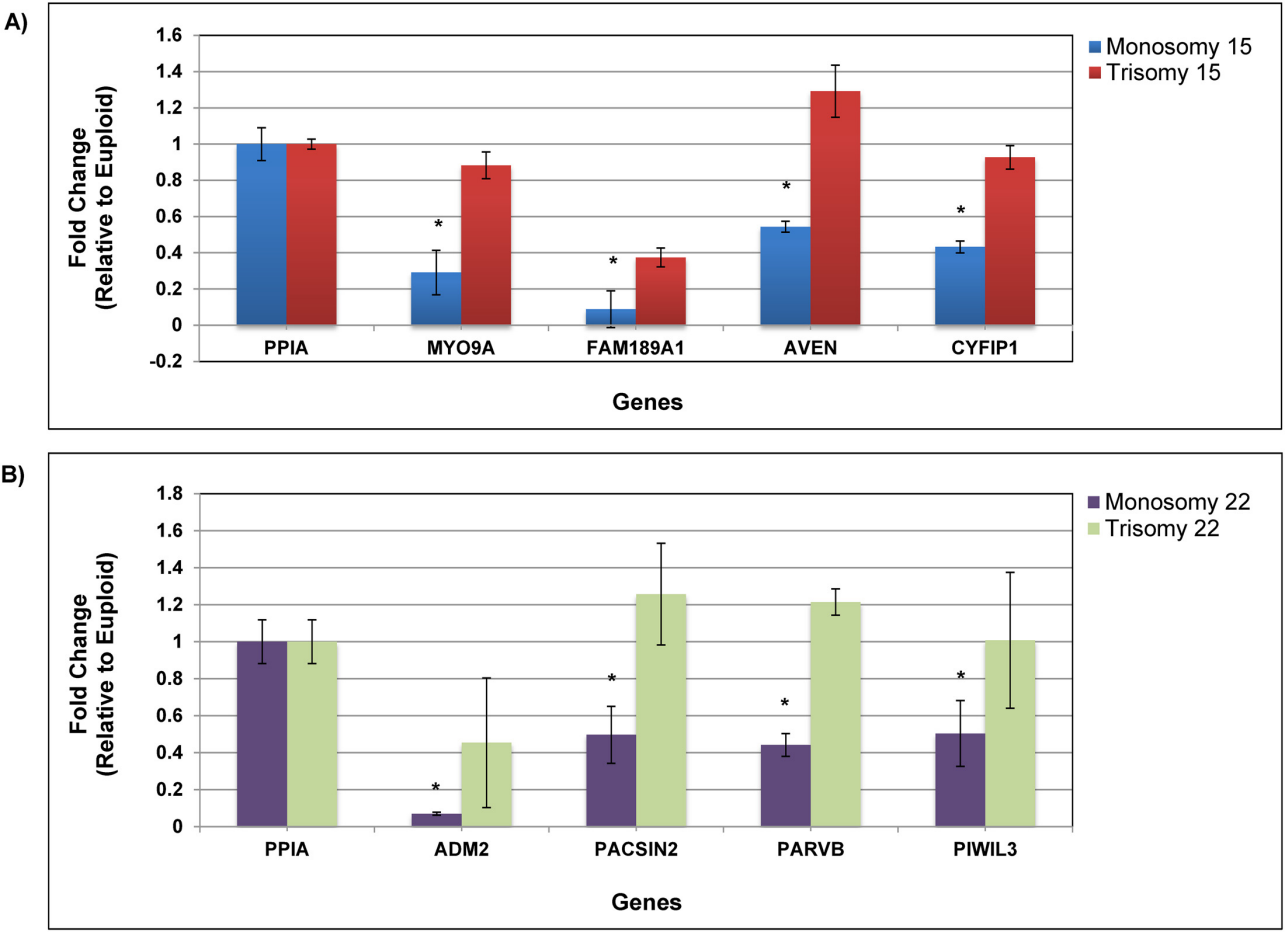
Developmental gene expression in individual human blastocysts (n = 10 replicates for each group) was performed by qPCR. Ct values were normalized to *PPIA*, an internal, constant housekeeping gene. Fold change was determined using the ΔΔCt method on the average of technical duplicates. Error bars represent standard error and the y-axis denotes fold change between euploid and aneuploidy. **A)** Signigicant decreased expression of chromosome 15 genes in monosomy 15 blastocysts compared to controls (*P < 0.05). **B)** Significant decreased expression of chromosome 22 genes in monosomy 22 blastocysts compared to controls (*P < 0.05).

mRNA analysis was performed on individual blastocysts that were monsomy or trisomy for chromosome 15, monosomy or trisomy for chromosome 22, and controls for key developmental genes located in cytoband regions with significantly altered methylation. The chromosome 15 genes *AVEN* (15q13.1), *CYFIP1* (15q11), *FAM189A1* (15q13.1) and *MYO9A* (15q22-q23) were all determined to have reduced expression in monosomy 15 blastocysts compared to control blastocysts (P < 0.05; Fig 1A). In contrast, trisomy 15 blastocysts had similar expression profiles to controls (ns). Chromosome 22 genes *ADM2* (22q13.33), *PACSIN2* (22q13.2-q13.33), *PARVB* (22q13.2-q13.33), and *PIWIL3* (22q11.23) were all shown to have significantly lower expression levels in monosomy 22 blastocysts compared to controls (P < 0.05; Fig 1B) with trisomy 22 blastocysts displaying no significant differences. All samples were normalized to the housekeeping gene, *PPIA*, which had stable expression within all sample groups.

**Fig 2:**
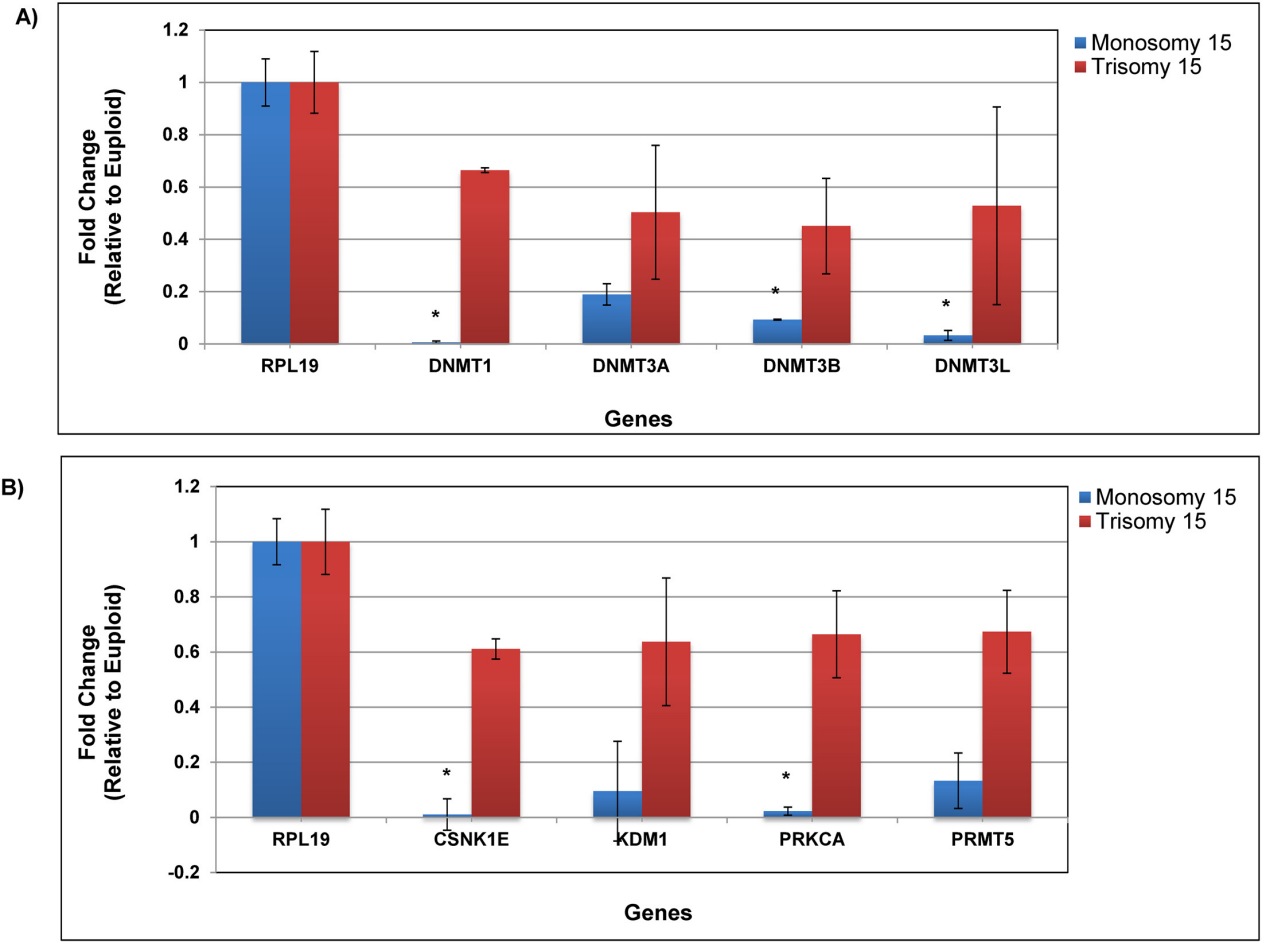
Epigenetic regulator expression in individual human blastocysts by qPCR (n = 12 replicates for each group). Ct values were normalized to *RPL19*, an internal, constant housekeeping gene. Fold change was determined using the ΔΔCt method on the average of technical duplicates. Error bars represent standard error and the y-axis denotes fold change between euploid and aneuploidy. **A)** The expression of DNA methylatransferases was analyzed in euploid, trisomy 15, and monosomy 15 blastocysts (*P < 0.05). **B)** The expression of post-translational regulators and the chromatin modifying protein, PRMT5 (*P < 0.05).

Gene expression analysis was also examined on additional, individual blastocysts for DNA methyltransferases and regulatory genes associated with establishing methylation. Monosomy 15 and trisomy 15 blastocysts were analyzed alongside controls for the following genes: *DNMT1*, *DNMT3A*, *DNMT3B*, *DNMT3L*, *CSNK1E*, *KDM1*, *PRKCA*, and *PRMT5*. It is important to note that none of these genes are located on chromosome 15 to avoid expression bias in these aneuploid samples. *RPL19* was used as the internal housekeeping gene and had constant, stable expression in all sample groups. All 4 DNA methyltransferases showed decreased expression in monsomy 15 blastocysts compared to controls (Fig 2A) with *DNMT1*, *DNMT3B*, and *DNMT3L* being statistically significant (P < 0.05). DNMT1 showed the largest difference with a nearly 10-fold decrease in expression observed in the monosomy 15 blastocysts. Post-translational regulatory genes responsible for the regulation of *DNMT1* gene expression (*CSNK1E*, *KDM1*, and *PRKCA*) revealed reduced expression in monosomy 15 blastocysts compared to controls (Fig 2B) with *CSNK1E*, *PRKCA*, and showing statistical significance (P < 0.05). The chromatin modifying protein, *PRMT5*, also displayed significantly decreased expression in monosomy 15 blastocysts (P < 0.05; Fig 2B). No expression differences were calculated to be statistically significant for any of the 8 genes examined between trisomy 15 and control blastocysts.

## Discussion

Chromosome segregation errors during maternal or paternal meiosis that lead to aneuploidy in the resulting embryo are well documented in human reproduction. While a handful of trisomy embryos (chromosomes 13, 18, 21, X and Y) can result in ongoing clinical pregnancies, monosomy embryos are rarely observed post-implantation, with Turner Syndrome being the only exception [19]. The bias against implantation of autosomal monosomies indicates that the lack of an autosomal chromosome is critical for development. This study investigated the relationship between chromosome aneuploidy epigenetic mechanisms and gene transcription as possible mechanisms to explain the low implantation potential of monosomy embryos.

Mammalian embryos undergo active and passive global demethylation, following fertilization, which reaches minimum levels at the morula/blastocyst stage. Therefore, unlike somatic cells with 50% methylation, the methylation status of human a blastocyst is significantly reduced [20]. Our results revealed a similar hypomethylated state of human blastocysts, independent of the blastocysts’ chromosome constitution (monosomy, trisomy, or diploid). Closer examination of the methylation profile of each individual chromosome revealed reduced methylation on the chromosome involved in the error for monosomy blastocysts. This could be reflective of the presence of only a single chromosome from the pair of chromosomes. In contrast, no methylation differences were observed between trisomy blastocysts and diploid controls, including for the extra chromosome involved in the aneuploidy. This observation could reflect dosage alterations of the trisomy blastocyst to normalize its transcriptome in order to offset the presence of the third chromosome [21]. In fact, evidence of this has been reported in studies of Down Syndrome that have shown tissue specific differences in the transcript levels of chromosome 21 genes [22, 23]. While there were genes that displayed an expected 50% increase in transcription, others exhibited no expression differences, and in some cases, even decreased expression was observed [22]. Transcriptional regulation in response to gene copy number for specific cell types could be the mechanism responsible for the observed gene dosage compensation [22]. Specifically, it has been suggested that stimulating mRNA degradation could be the active mechanism that allows for post-transcriptional buffering of aneuploidy in trisomy cells [24].

To determine if the methylation changes observed on the chromosome associated with the error were disrupting gene transcription, mRNA analysis was performed on key developmental genes. *AVEN*, *CYFIP1*, *FAM189A1*, and *MYO9A* are located within specific cytogenic regions of chromosome 15 that displayed the most substantial levels of hypomethylation in monosomy 15 blastocysts. Transcriptional analysis revealed significant reduction in expression compared to controls for these developmental genes. *AVEN* plays an important role in male and female germ cell development and has been shown to induce apoptosis in cells that have large amounts of DNA damage [25]. Reduced expression would diminish the apoptotic activity required to prevent abnormal cells from further development, thereby allowing these monosomy embryos to progress further than they should, forcing their demise prior to implantation. *CYFIP1* is involved in mRNA translation and knockout mouse embryos have been shown to be significantly reduced in size, developmentally delayed, and do not survive past the blastocyst stage [26, 27]. This has important implications for monosomy embryos. Although they can grow to the blastocyst stage and appear to be of good quality, suitable expression levels of *CYFIP1* are essential for further embryonic development and proper implantation. *FAM189A1* is a CD20-like multi-pass transmembrane protein that is required for cell signaling [28]. These proteins are expressed on the surface of B-cells which are important for antibody response. With pregnancy being a pro-inflammatory state, proper expression of these proteins would be required for successful implantation to occur. *MYO9A* mutations are known to cause several diseases in humans [29]. This gene is a class IX myosin molecule that is important for epithelial formation and downregulation of *MYO9A* has been shown to affect cell morphology and differentiation [30]. Complete knockdown disrupts the formation and stabilization of cell-to-cell contacts during early development. Reduced expression of *MYO9A* could be greatly impacting the ability of monosomy blastocysts to have functional interactions with the uterus, thereby reducing the ability to implant and develop into a viable pregnancy.

*ADM2*, *PACSIN2*, *PARVB*, and *PIWIL3* are located within highly significant hypo-methylated cytogenic regions of chromosome 22 and were all found to have significantly lower expression in monosomy 22 blastocysts compared to controls. *ADM2* is an invasion promoting peptide that regulates placental mucin 1 (*MUC1*) and plays an important role in embryo implantation by promoting placental growth and inhibiting *MUC1* expression in order to assist in trophoblast invasion [31]. *PACSIN2* plays a role in endocytosis [32] and cell migration [33]. Decreases in *PACSIN2* expression have been postulated to result in unregulated activation of α5β1 integrin which would reduce the ability of mesodermal cells to migrate [34]. This would have a very severe impact on the ability of a monosomy embryo to implant. *PARVB* is involved in cell adhesion and survival and also plays an important role in angiogenesis which promotes tumor growth in cancers [35–37]. The biology of tumor development and progression is similar to that of trophoblast invasion required for implantation. Reduced expression would prevent these cells from sufficiently being able to invade the maternal uterus. Likewise, *PIWI* genes are mainly expressed in germ cells and their proteins participate in germ cell differentiation with overexpression leading to malignancy [37]. *PIWIL3*, specifically, is required for early mammalian oogenesis and embryogenesis [38] and the under expression observed in monosomy blastocysts, again, could prevent trophoblast invasion leading to failed implantation.

Each of these developmental genes on chromosomes 15 and 22 displayed, roughly, a 0.5-fold expression decrease in monosomy blastocysts and could be contributing to their overall reduced competence and lack of implantation potential. Gene dosage is likely a contributing factor for this reduced expression, with the presence of only a single chromosome from the pair of chromosomes. In contrast, the transcription levels for each of the developmental genes in trisomy 15 and trisomy 22 blastocysts remained unchanged compared to controls. This indicates transcriptional compensation by trisomy embryos, away from the expected 1.5-fold increase, which could explain their future implantation potential.

Additional mRNA analysis was performed to determine if the processes involved in establishing methylation are impacted in monosomy blastocysts. DNA methyltransferases are the enzymes responsible for DNA methylation acquisition and maintenance during embryogenesis. *DNMT1* is the maintenance methyltransferase that replicates methylation patterns on daughter DNA strands during mitosis [39]. *DNMT3A*, *3B*, *and 3L* are *de novo* methyltransferases that set up DNA methylation patterns early in embryonic development, initiating at the blastocyst stage, and are also required for establishing maternal genomic imprints in gametes [14]. *DNMT1*, *DNMT3B*, and *DNMT3L* all displayed significantly reduced expression in monosomy blastocysts compared to either controls or trisomy blastocysts.

Reduced transcription was also observed in monosomy blastocysts for two post-translational regulatory genes, *CSNK1E* and *PRKCA*, which are required for *DNMT1* activity. These two genes showed no differences when comparing expression profiles between trisomy blastocysts and controls. Furthermore, reduced gene expression was confirmed only in monosomy blastocysts for a chromatin modifying protein, *PRMT5*, which is recruited along with the *DNMTs*, to remodel histones through arginine methylation, resulting in the silencing of genes [40]. *PRMT5* has been shown to be required throughout the resetting of the epigenome, during preimplantation development [41]. These combined mRNA expression data in monosomy blastocysts compared to trisomy or controls suggest that a decrease in the functionality of *DNMT* machinery may result during cell division and DNA replication due to the presence of only a single chromosome from the pair, thereby compromising further development.

In conclusion, this novel study revealed hypomethylation of the chromosome involved in the error for monosomy blastocysts, alongside decreased expression of developmental genes located on the chromosome of error and altered transcription of DNA methylation processes. Taken together, the altered methylation and disrupted downstream transcription could be directly impacting the developmental and implantation potential of monosomy blastocysts as it is well known that the autosomal monosomy state of a whole chromosome is not well tolerated during the window of implantation. In contrast, the trisomy blastocyst displays transcriptional dosage compensatory mechanisms for the presence of an additional chromosome, revealing similar methylation and gene expression to controls, and thereby giving an explanation for the difference in the implantation potential between trisomy and monosomy embryos. Future studies investigating epigenetic mechanisms associated with chromosome constitution may further expand our knowledge of human chromosomal aneuploidy and increase our understanding of its origins and impact during the window of implantation.
